# Supplementary material for: Methylotrophic yeast Candida boidinii enhances the colonization of plant growth-promoting yeast Papiliotrema laurentii in the phyllosphere
Source: Front Microbiol. 2025 Nov 21;16:1677102. doi: 10.3389/fmicb.2025.1677102 (PMC12679942; doi:10.3389/fmicb.2025.1677102)
Supplement: Supplementary file 2 [file Data_Sheet_1.pdf]

**Supplementary Information**

**Methylotrophic yeast *Candida boidinii* enhances the colonization of plant growth-promoting yeast *Papiliotrema laurentii* in the phyllosphere**

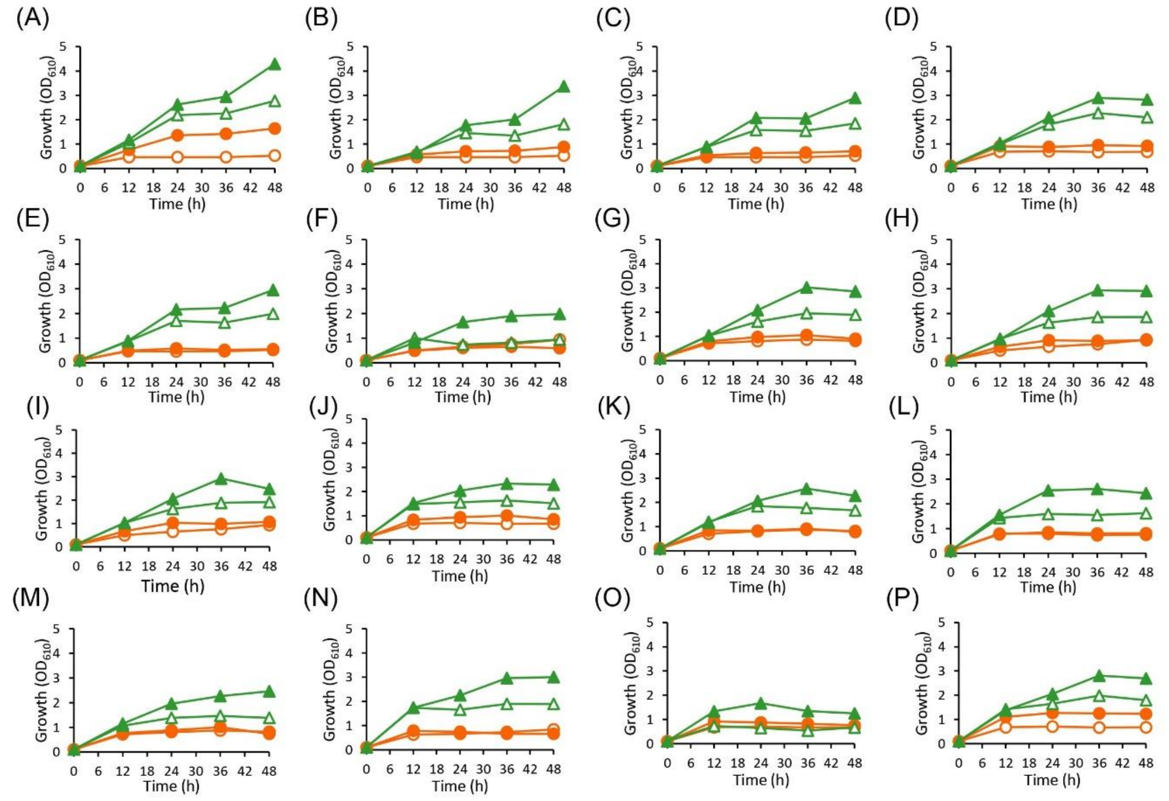


**Supplemental Fig. 1 Cultivation of the other representative yeast strains with *C. boidinii* strain AOU-1 in SP liquid media using *Beppu* flasks.**

Growth curve of *C. boidinii* strain AOU-1 and (A) *A. melanogenum* strain GiL12, (B) *Cryptococcus* sp. strain FCL26, (C) *C. calyptogenae* strain PeF7, (D) *C. minutum* strain GSMF2, (E) *C. slooffiae* strain GiL13, (F) *M. caribbica* strain GoKF3, (G) *N. diffluens* strain ApF1, (H) *P. aurea* strain FCL21, (I) *P. kluyveri* strain GoKF5, (J) *P. hubeiensis* strain SAL1, (K) *P. pruni* strain FCL20, (L) *R. ruineniae* strain GiL14, (M) *R. paludigena* strain GSMF1, (N) *R. toruloides* strain PeF8, (O) *S. intermedia* strain GoKF2, and (P) *Z. obscura* strain GFF1. Symbols: (empty circles) *C. boidinii* strain AOU-1 mono-cultivation; (filled circles) *C. boidinii* strain AOU-1 co-cultivation; (empty triangle) each representative yeast strain mono-cultivation*;* (filled triangle) each representative yeast strain co-cultivation.

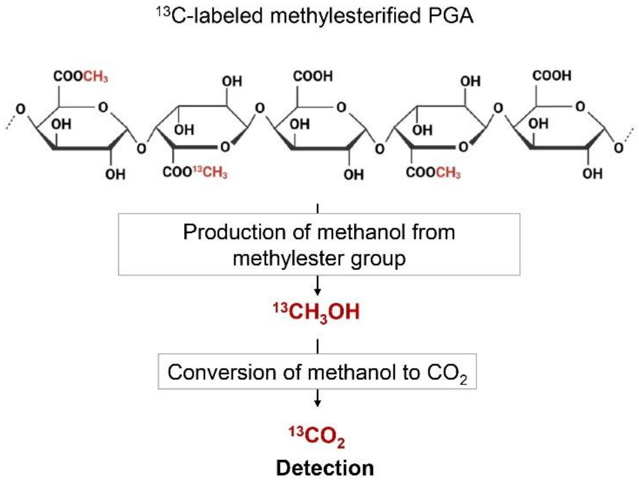


**Supplemental Fig. 2 Tracing $^{13}$C-labeled $CO_2$ formation from the methylester group of pectin.** Hydrolysis of the $^{13}$C-labeled methylester group of pectin releases $^{13}$C-enriched methanol, which is converted to $^{13}$C-enriched $CO_2$, detectable as a final product by gas chromatography-isotope-ratio-mass spectrometry (GC-IRMS).

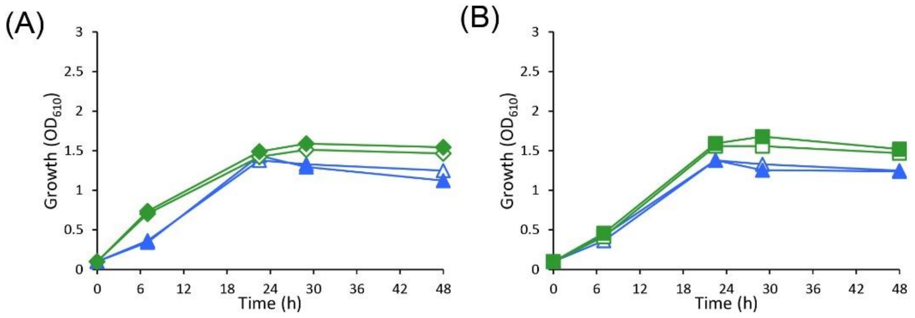


**Supplemental Fig. 3 Cultivation of *P. laurentii* strain PeF4 with *R. ruineniae* strain GiL14 and *R. toruloides* strain PeF8 in SP liquid media using *Beppu* flasks.**
Growth curve of *P. laurentii* strain PeF4 and (A) *R. ruineniae* strain GiL14, and (B) *R. toruloides* strain PeF8, during mono- and co-cultivation. Symbols: (empty triangle) *P. laurentii* strain PeF4 mono-cultivation*;* (filled triangle) *P. laurentii* strain PeF4 co-cultivation; (empty diamonds) *R. ruineniae* strain GiL14 mono-cultivation; (filled diamonds) *R. ruineniae* strain GiL14 co-cultivation;.(empty squares) *R. toruloides* strain PeF8 mono-cultivation; (filled squares) *R. toruloides* strain PeF8 co-cultivation.

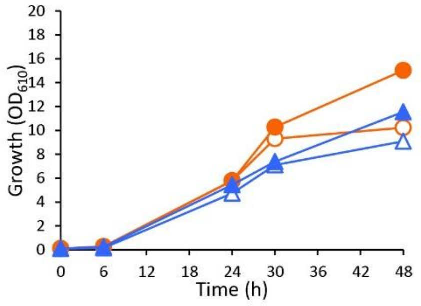


**Supplemental Fig. 4 Cultivation of *P. laurentii* strain PeF4 with *C. boidinii* strain AOU-1 in SD liquid media using *Beppu* flasks.**

Growth curve of *C. boidinii* strain AOU-1 and *P. laurentii* strain PeF4 during mono- and co-cultivation. Symbols: (empty circles) *C. boidinii* strain AOU-1 mono-cultivation; (filled circles) *C. boidinii* strain AOU-1 co-cultivation; (empty triangle) *P. laurentii* strain PeF4 mono-cultivation*;* (filled triangle) *P. laurentii* strain PeF4 co-cultivation.

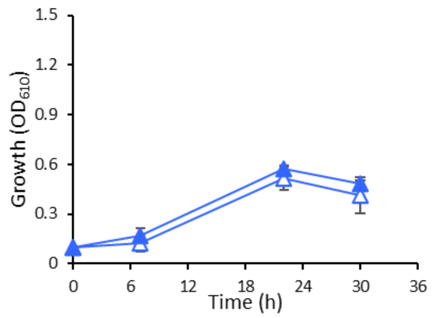


**Supplemental Fig. 5 Growth curve *P. laurentii* strain PeF4 in SP and SPM liquid media.**

Symbols: (empty triangles) *P. laurentii* strain PeF4 in SP medium; (filled triangles) *P. laurentii* strain PeF4 in SPM liquid medium.

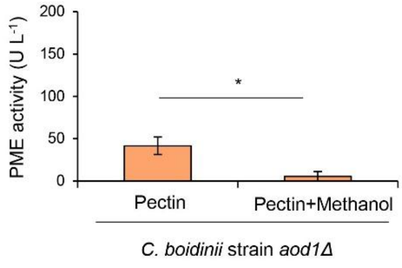


**Supplemental Fig. 6 Specific extracellular PME activity of *C. boidinii* strain *aod1Δ* in SP and SPM liquid media.**

The data are presented as the mean±standard error (SE; n = 6). Significant differences are marked by *($p < 0.05$; Student's *t*-test).

**Tables**

**Supplemental Table 1: The representative yeast strains isolated and identified from the phyllosphere**
